# Genomic Analysis of Sin Nombre Virus Sequences, Northwestern United States, 2023

**DOI:** 10.3201/eid3205.251476

**Published:** 2026-05

**Authors:** Grant Rickard, Ricardo Rivero, A. Catherine Grady, Jennifer Horton, Cody J. Lauritsen, Stephen Fawcett, Samuel M. Goodfellow, Hanna N. Oltean, M. Pilar Fernandez, Stephanie N. Seifert

**Affiliations:** University of Washington School of Medicine, Seattle, Washington, USA (G. Rickard); Washington State University Paul G. Allen School for Global Health, Pullman, Washington, USA (G. Rickard, R. Rivero, A.C. Grady, J. Horton, C.J. Lauritsen, S. Fawcett, M.P. Fernandez, S.N. Seifert); Children’s Hospital Los Angeles, Los Angeles, California, USA (S.M. Goodfellow); Washington State Department of Health Zoonotic and Vector-borne Disease Program, Tumwater, Washington, USA (H.N. Oltean)

**Keywords:** Orthohantavirus sinnombreense, Sin Nombre virus, viruses, zoonoses, Washington, Idaho, United States

## Abstract

We report Sin Nombre virus (SNV) genome sequences in the northwestern United States, including SNV sequences recovered from montane voles. Analysis of samples collected from 189 individual rodents revealed high SNV prevalence in the region and evidence of virus reassortment or coinfection, highlighting ongoing virus diversification in rodents.

Sin Nombre virus (SNV; *Orthohantavirus sinnombreense*), is a member of the family Hantaviridae and the primary cause of hantavirus pulmonary syndrome in North America. First identified during a 1993 outbreak in the Four Corners region of the United States, SNV is linked to severe respiratory disease and high mortality rates (*1*). During 1993–2022, a total of 864 hantavirus pulmonary syndrome cases were reported in the United States, with a 36% case-fatality rate (*2*,*3*); 109 of those cases occurred in the northwestern states of Idaho, Oregon, and Washington.

SNV primarily is maintained by *Peromyscus* spp. deer mice, widespread rodents that are frequently associated with agricultural and peridomestic settings. Human infection usually results from inhalation of aerosolized virus particles from contaminated excreta (*4*,*5*), and zoonotic risk is influenced by ecologic factors (*6*). Although SNV commonly is detected in deer mice, several reported detections in sympatric rodent species, and broad geographic distributions suggest greater complexity in hantavirus maintenance (*7*–*9*).

 Virus genomic surveillance can provide insights on virus evolution and spread (*10*), but <100 full SNV genomes have been published, none of which are from the northwestern United States (*3*). We report detection and genome sequences of SNV in the northwestern United States in montane voles and western deer mice.

## The Study

**Figure 1:**
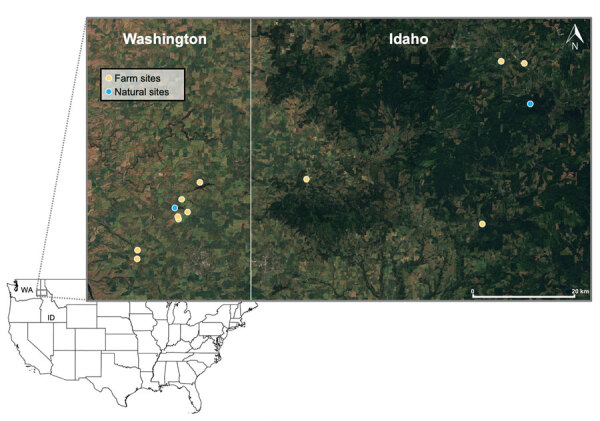
Locations of successful unique rodent sampling grids, by location type, in a study of Sin Nombre virus in rodent species sampled from farms and natural sites, Palouse region, eastern Washington and western Idaho, USA, 2023. Inset map indicates location of study area relative to rest of United States. Basemap generated in Sentinel-2 Cloudless (EOX IT Services, https://eox.at). State boundary based on US Census Bureau cartographic boundary files (https://www.census.gov/geographies/mapping-files/time-series/geo/carto-boundary-file.html).

During June–August 2023, we live-trapped rodents at farms and natural areas in the Palouse region of eastern Washington and western Idaho (Figure 1), a major agricultural hub dominated by wheat and canola fields. We conducted sampling over 3 consecutive nights, including repeated fecal sampling with mark-recapture and lethal collection on the third night. We collected and tested samples from 189 rodents across agricultural and natural landscapes in the Palouse. We identified species using morphologic criteria in consultation with regional experts. We deployed Sherman live traps at 8 farms and 2 forested sites in 2 grids (100 m × 30 m) at each location. All procedures followed American Veterinary Medical Association guidelines and were approved by the Washington State University Institutional Animal Care and Use Committee (Animal Study Approval Form no. 6927). We performed sampling under Idaho scientific collections permits 36112 and JOE1 and Washington state scientific collections permit SEIFERT 23–122.

We collected fecal samples on all trapping nights, in addition to serum, lung tissue, and bladder tissue on the third and final trapping night at each site. We report only data from the final capture for each individual rodent sampled. Across all sites, we collected samples from 2 creeping voles (*Microtus oregoni*), 18 montane voles (*Microtus montanus*), 4 western meadow voles (*Microtus drummondii*), 2 house mice (*Mus musculus*), 153 western deer mice (*Peromyscus sonoriensis*), 1 yellow pine chipmunk (*Neotamias amoenus*), and 9 least chipmunks (*Neotamias minimus*). 

We used SNV nucleocapsid protein (BEI Resources, https://www.beiresources.org) to assess seroreactivity in rodent serum samples. We immobilized the nucleocapsid protein on Maxisorp-plates (Thermo Fisher Scientific, https://www.thermofisher.com) and applied serum diluted 1:100. We detected nucleocapsid protein bound antibodies with horseradish peroxidase-conjugated goat anti-rat secondary antibody (AbCAM, https://www.abcam.com). We assessed seropositivity for each plate as the mean optical density of 4 naive serum controls +3 SD.

We extracted total RNA from tissue samples and fecal samples by using the Zymo Quick-RNA MagBead kit (Zymo Research, https://www.zymoresearch.com), according to the manufacturer’s protocol for each sample type. We detected presence of virus RNA by using quantitative reverse transcription PCR (qRT-PCR), as previously described (*11*), with an internal control targeting the *P. maniculatus* hypoxanthine-guanine phosphoribosyltransferase gene to monitor extraction and amplification performance with in-house–designed forward primer (5′-CAAAGCCTAAGAGGAGAGTTCA-3′), reverse primer (5′-GATGGCCGCAGAACTAGAA-3′), and probe (5HEX/AGGAGTCCC/ZEN/ATTGATGTTGCCAGT/3IABkFQ).

Western deer mice demonstrated high prevalence by serologic testing (26%) and qRT-PCR (9.8%) across all sites (Table). Among qRT-PCR–positive western deer mice, lung cycle threshold (Ct) values ranged from 18.1 to 34.1 (mean 25.3). Two adult male western deer mice had qRT-PCR–positive bladder samples with Ct values of 31.9 and 33.6; both rodents had low lung Ct values (18.1 and 21.5). Montane voles on farmlands showed the highest prevalence, having 50% seroprevalence and 22.2% qRT-PCR–positive lung tissue samples (Table) and Ct values of 29.5 and 26.5, which were within the range observed in western deer mice. We note recent findings in New Mexico showing similarly high prevalence for SNV in diverse rodent taxa, including recovery of infectious virus (*9*). Fewer lung samples were qRT-PCR–positive than seropositive rodents. We deposited surveillance data for this study in the Pathogen Harmonized Surveillance database (https://pharos.viralemergence.org/projects/?prj = prjdGTXI9IIBo).

**Table:** Serologic and quantitative reverse transcription PCR results for Sin Nombre virus in rodent species sampled from farm sites and natural sites, Palouse region, eastern Washington and western Idaho, USA, 2023*

Site and species	Age category/sex	Sample type, no. (%)			
		Blood	Lung	Bladder	Fecal
Farm site					
Western meadow vole (*Microtus drummondii*)	Adult/F	ND	0/1	0/1	0/1
	Adult/M	1/1	0/2	0/1	0/1
	Juvenile/M	0/1	ND	0/1	0/1
	Total	1/2 (50)	0/3	0/3	0/3
Montane vole (*Microtus montanus*)	Adult/F	0/2	0/2	0/2	0/4
	Adult/M	3/3	1/3	0/3	0/5
	Juvenile/F	0/1	0/1	0/1	0/4
	Juvenile/M	1/2	1/3	0/3	0/4
	Total	4/8 (50)	2/9 (22)	0/9	0/17
Creeping vole (*Microtus oregonii*)	Juvenile/F	ND	0/1	0/1	0/1
	Juvenile/M	0/1	0/1	0/1	0/1
	Total	0/1	0/2	0/2	0/2
House mouse (*Mus musculus*)	Adult/M	ND	ND	ND	0/1
	Juvenile/F	ND	ND	ND	0/1
	Total	ND	ND	ND	0/2
Yellow-pine chipmunk (*Neotamias amoenus*)	Adult/F	ND	ND	ND	0/1
Least chipmunk (*Neotamias minimus*)	Adult/F	0/1	0/1	0/1	0/2
	Adult/M	ND	ND	ND	0/1
	Juvenile/F	0/1	0/1	0/1	0/1
	Juvenile/M	1/2	0/1	0/2	0/3
	Total	1/4 (25)	0/3	0/4	0/7
Western deer mouse (*Peromyscus sonoriensis*)	Adult/F	5/25	1/27	0/27	0/35
	Adult/M	11/28	7/28	2/27	0/35
	Juvenile/F	6/15	0/16	0/16	0/24
	Juvenile/M	2/14	1/14	0/14	0/15
	Total	24/82 (29)	9/85 (11)	2/84 (2)	0/109
Natural site					
Montane vole (*Microtus montanus*)	Adult/F	ND	0/1	0/1	0/1
Least chipmunk (*Neotamias minimus*)	Adult/F	0/1	0/1	0/1	0/1
	Adult/M	0/1	0/1	0/1	0/1
	Total	0/2	0/2	0/2	0/2
Western deer mouse (*Peromyscus sonoriensis*)	Adult/F	2/8	0/10	0/10	0/12
	Adult/M	3/9	2/11	0/11	0/14
	Juvenile/F	0/7	0/7	0/7	0/8
	Juvenile/M	1/10	0/10	0/10	0/10
	Total	6/34 (18)	2/38 (5)	0/38	0/44

We modeled SNV lung positivity detected by qRT-PCR as a binary outcome by using logistic regression with mean bias reduction in the R 4.1.3 brglm2 package (The R Project for Statistical Computing, https://www.r-project.org) for species with qRT-PCR–positive lung tissue samples. Predictors were land type (forest or farm), sex, age, and species (*M. montanus* montane voles or *P. sonoriensis* western deer mice). Male rodents had significantly higher odds of qRT-PCR positivity in lung tissue samples than did female rodents (odds ratio 9.42 [95% CI 1.76–50.5]), whereas land type, species, and age were not significant predictors. Elevated SNV prevalence in male deer mice aligns with known SNV ecology, although the precise mechanism is unknown (*12*). 

We sequenced SNV by using a tiled amplicon scheme proposed by Goodfellow et al. (*13*) on the Oxford Nanopore platform (https://nanoporetech.com). We quality controlled reads, trimmed primers, mapped reads, and extracted the consensus sequence by using an in-house assembly pipeline (https://github.com/viralemergence/SNVler). We recovered sequence data for all 3 SNV genome segments from 10 individual rodents, including 2 montane voles; segment completeness ranged from 24% to 100% and depth ranged from 6.8 to 476.1 times (GenBank accession nos. PX401008–37). To address persistent medium-segment dropouts, we designed flanking primers (MsegFor 5′-GCAGGTAGCTGATCTCAAG-3′ and MsegR 5′-CCAGTCCATGTAAGAGGTAC-3′) for amplification and sequencing, improving assemblies and guiding future refinement of the primer set for SNV in the northwestern United States.

**Figure 2:**
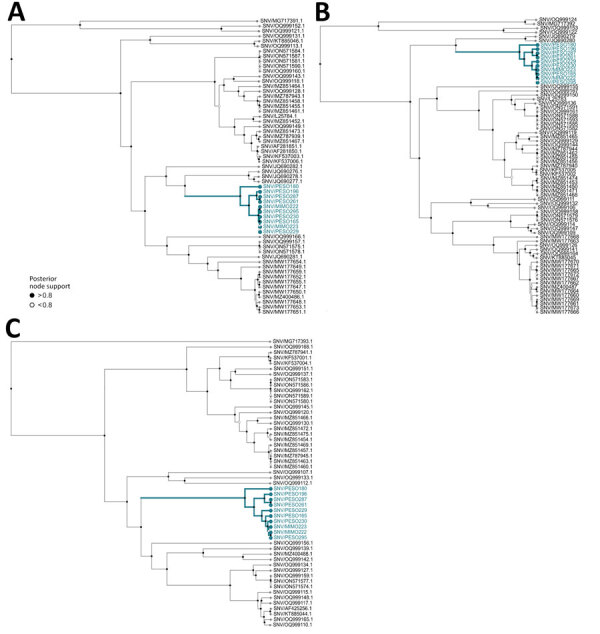
Phylogenetic reconstruction of samples from Washington, USA, of Sin Nombre virus in context of virus diversity in the United States, 2023. A) Small segment; B) medium segment; C) large segment. Blue highlighting indicates clade grouping of Washington samples generated in this study. MIMO indicates sequences from *Microtus montanus* montane voles. PESO indicates sequences from *Peromyscus sonoriensis* western deer mice.

We analyzed segment-wise alignments curated from GenBank in BEAST 1.10.5 (https://beast.community) (strict clock, exponential coalescent), using uniform tip sampling for incomplete collection dates (*14*). Estimated clock rates were 1.19 × 10^−4^, 1.203 × 10^−4^, and 1.223 × 10^−4^ substitutions/site/year; root-to-tip regression resulted in R^2^ values of 0.197 (p value 4.16 × 10^–04^) for the small segment, 0.067 (p-value 0.0282) for the medium segment, and 0.257 (p value 1.056 × 10^–04^) for the large segment, suggesting a weak temporal signal. This pattern probably reflects confounding between sampling time and geographic structure, underscoring the need for broader spatial and temporal sequencing to resolve the SNV phylogeny. Palouse sequences formed a distinct clade closest to SNV genomes from Montana collected during 2008–2009 (*11*) (Figure 2).

**Figure 3:**
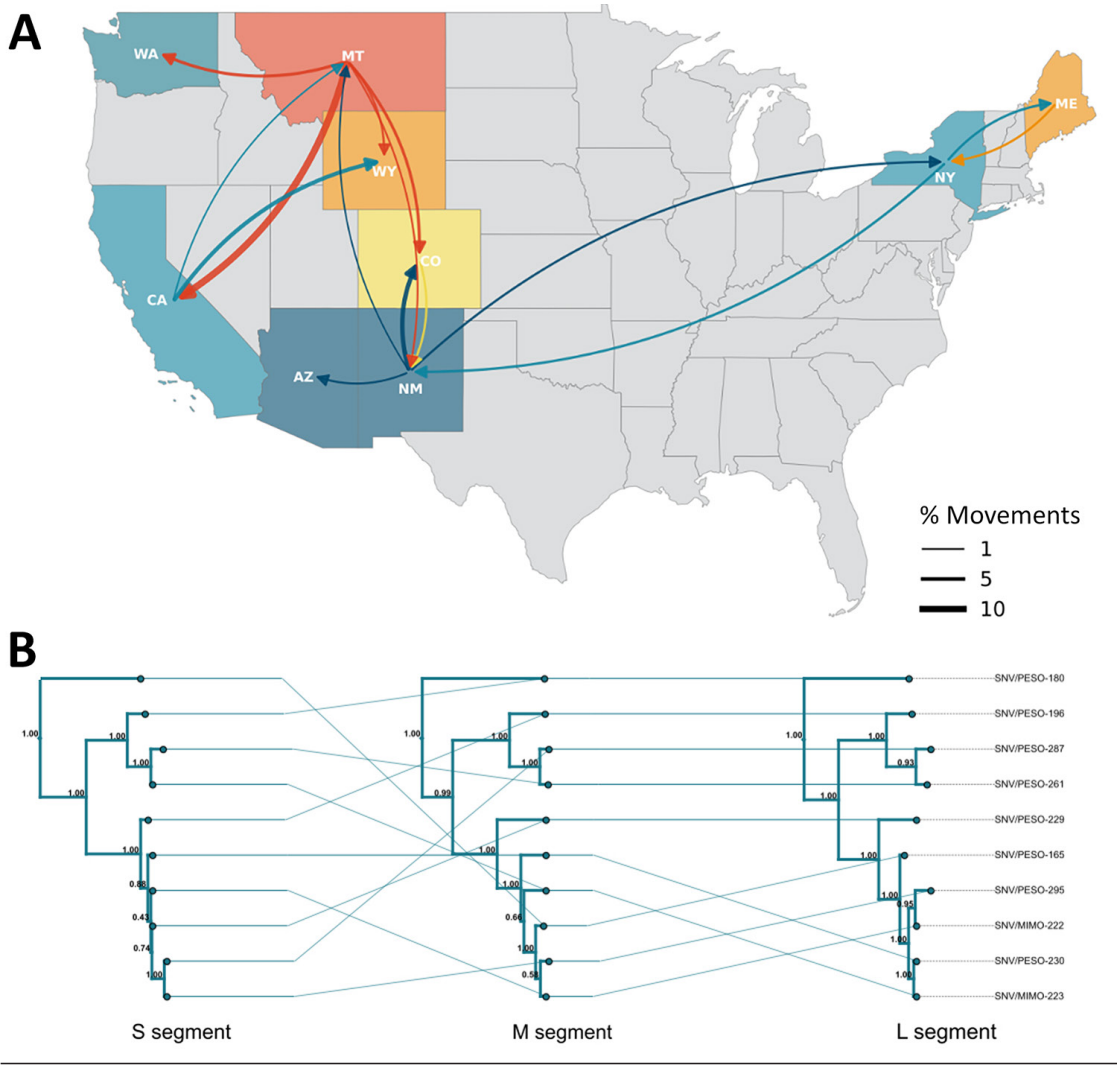
Evolutionary dynamics of Sin Nombre virus sequences, Washington, USA, 2023. A) Phylogeographic reconstruction of movements between discrete states using Markov jumps. Curve weight reflects Bayes factor support. B) Tanglegram showing topologic changes across segment trees. Connecting lines link variants. Major shifts indicate reassortment. Posterior node support shown at each node. L, large; M, medium; S, small.

Discrete phylogeography based on the small segment tree inferred introduction into Washington from Montana circa 1915 (95% highest posterior density 1873–1982), followed by local diversification (Figure 3, panel A). Bayesian stochastic search variable selection analysis (*15*) shows low support for Montana–Washington movement, suggesting unsampled intermediates and illustrating the need for improved genomic surveillance. Topologic discordance among viral segments (Figure 3, panel B) supports reassortment or coinfection with segment-specific differences in within-host abundance that could influence consensus recovery. Local variants from western deer mice and montane voles cluster with high support, suggesting cross-species transmission between rodents in the northwestern United States (Figure 3, panel A).

## Conclusions

We report SNV genome sequences from the northwestern United States, addressing a longstanding regional data gap. Our findings indicate reassortment or co-infection among sympatric rodent hosts, underscoring the complexity of SNV evolution and maintenance. Developing targeted primers to overcome chronic regional dropout enabled recovery of key genomic regions and will guide efforts to recover SNV genomes in this understudied region. Clarifying how multiple hosts contribute to virus exchange will improve understanding of transmission dynamics and zoonotic risk.
